# Supplementary figures and images for: ATG5 is instrumental in the transition from autophagy to apoptosis during the degeneration of tick salivary glands
Source: PLoS Negl Trop Dis. 2021 Jan 29;15(1):e0009074. doi: 10.1371/journal.pntd.0009074 (PMC7875341; doi:10.1371/journal.pntd.0009074)

## Fig1A


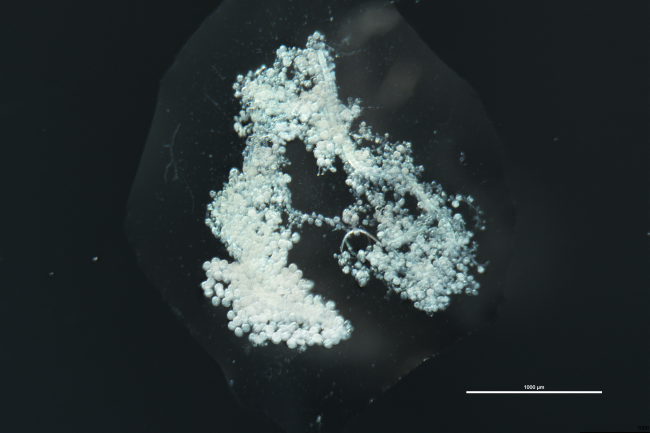

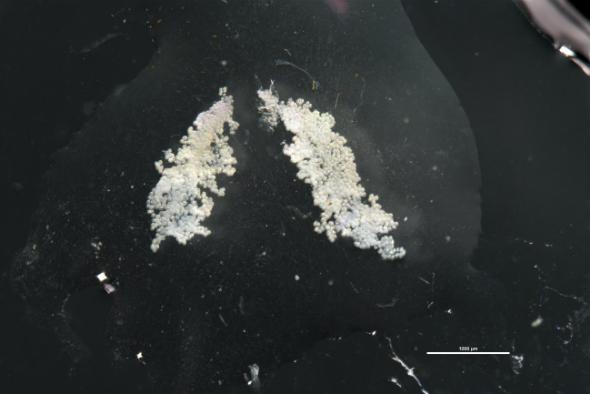

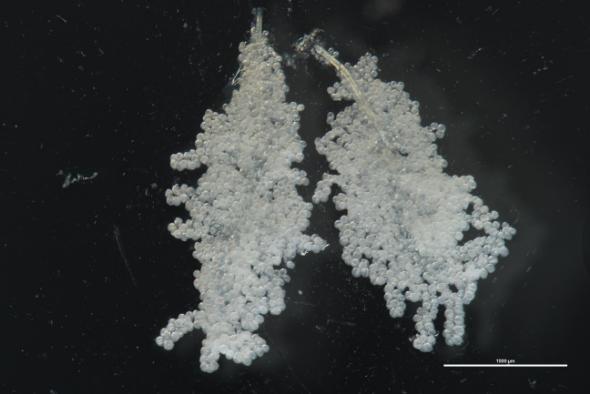

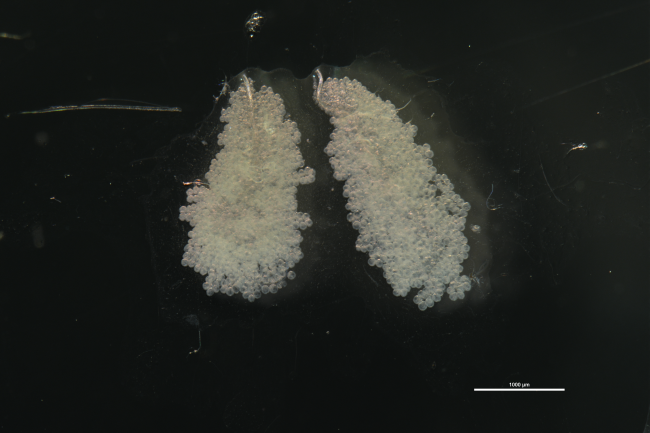

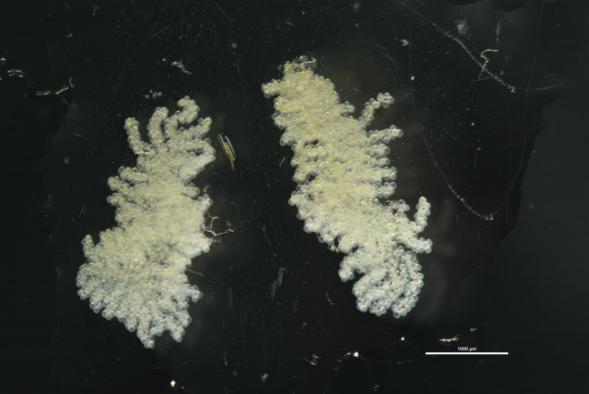

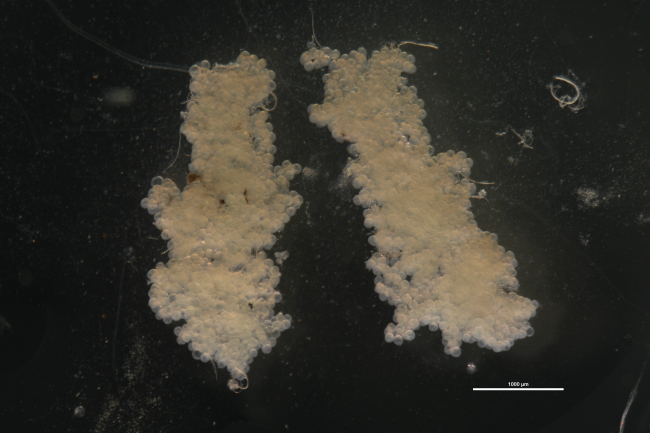

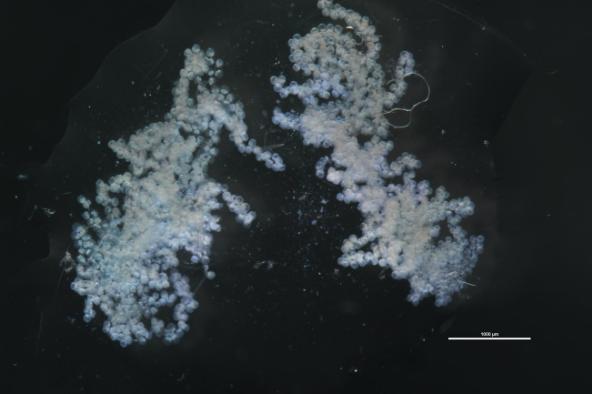

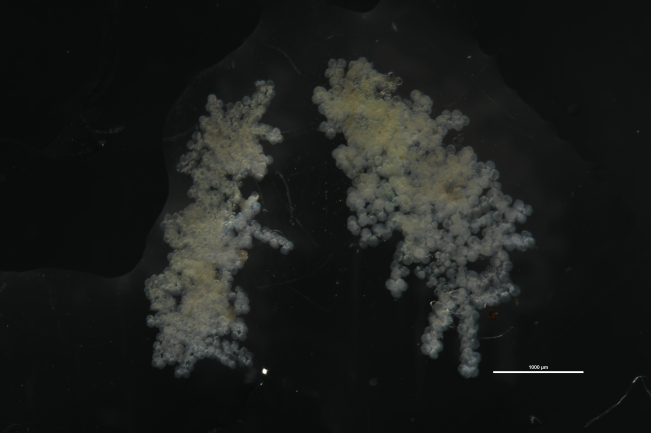

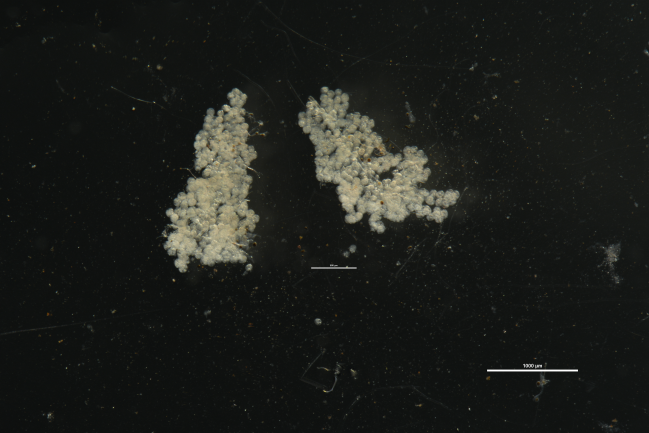


## Fig1C


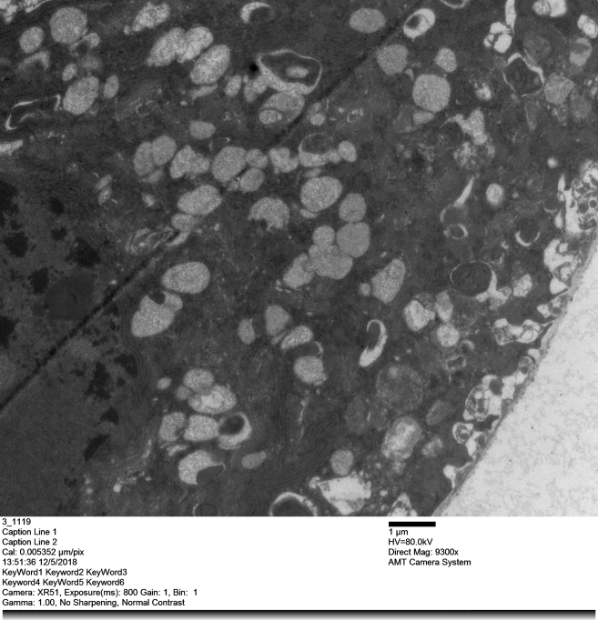

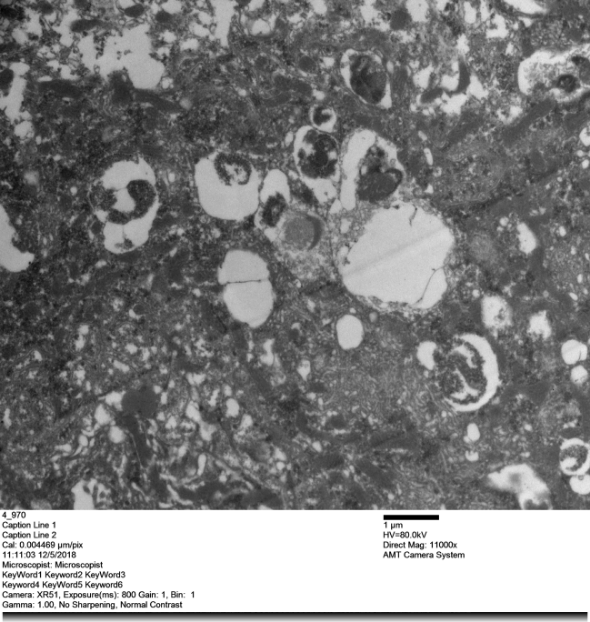


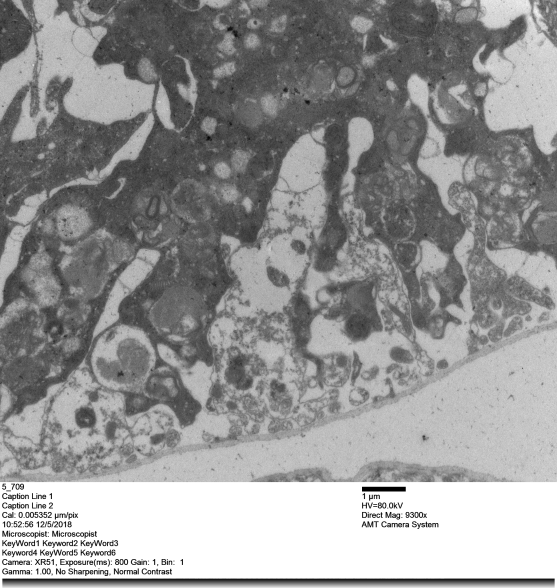

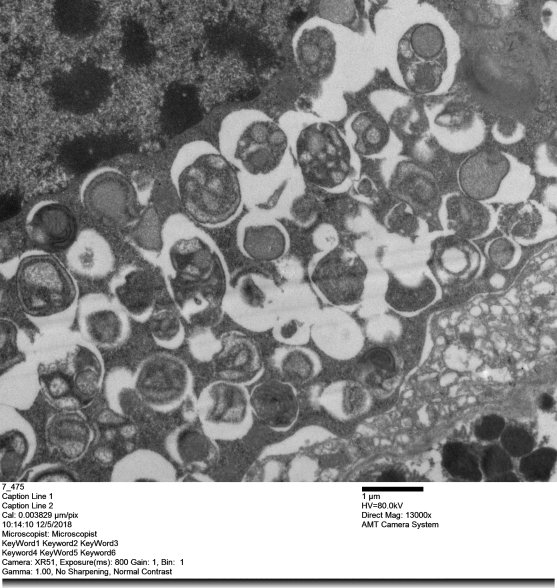


## Fig1D


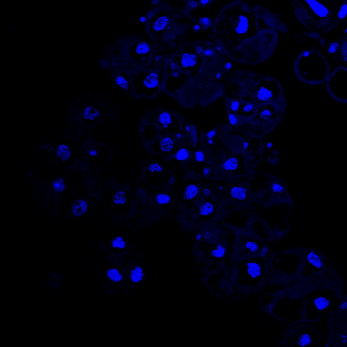

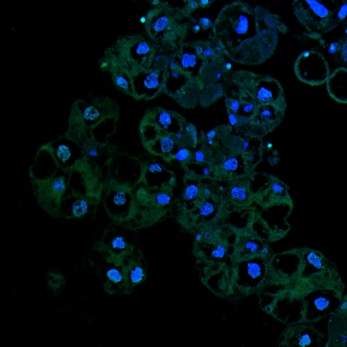

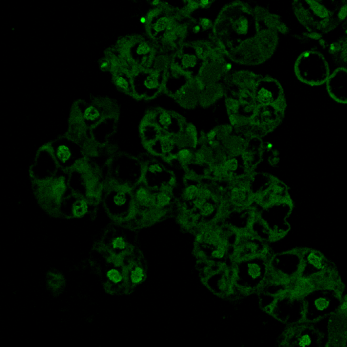


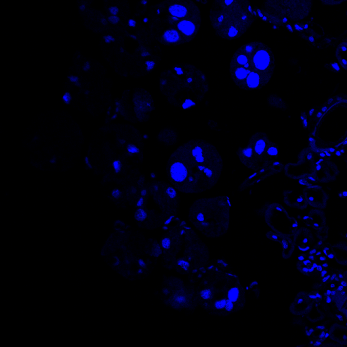

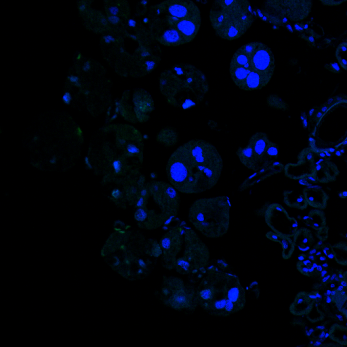

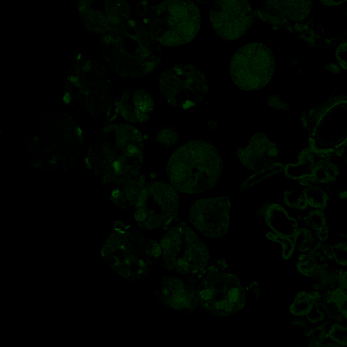


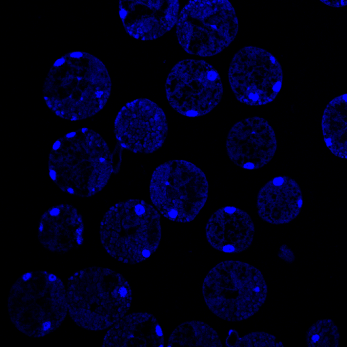

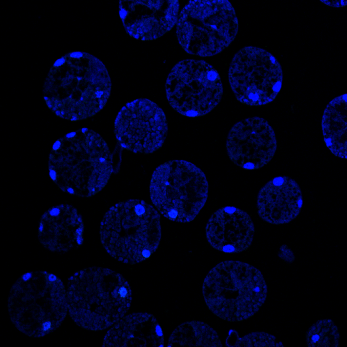

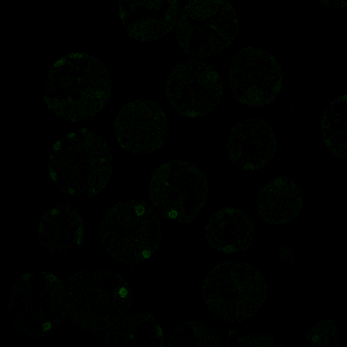


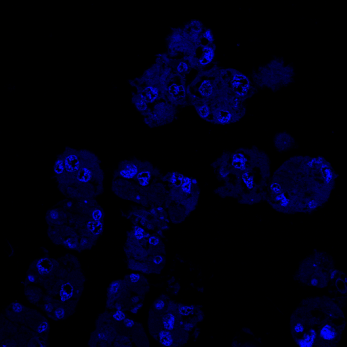

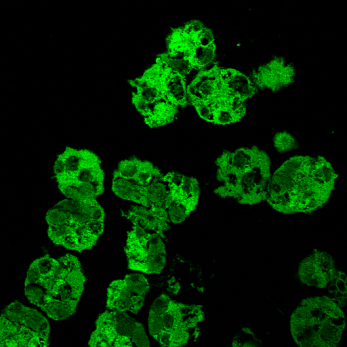

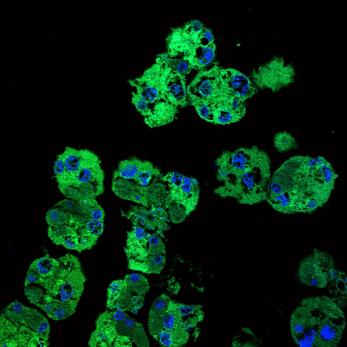


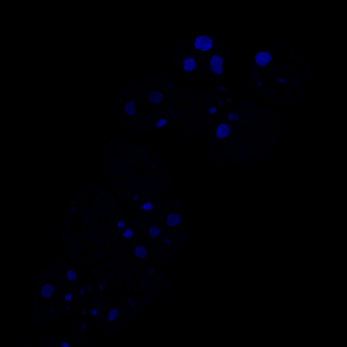

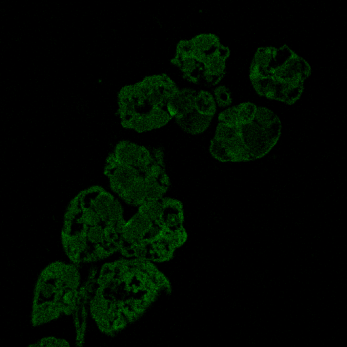

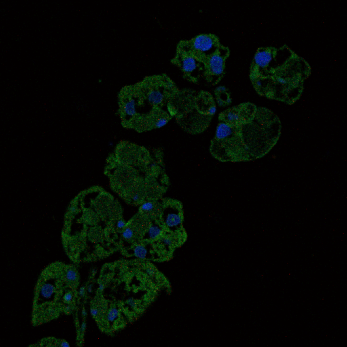


## Fig3A


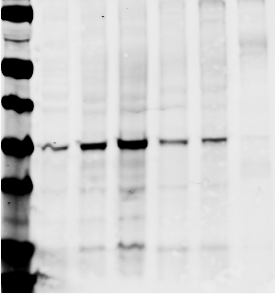


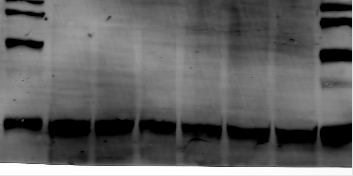


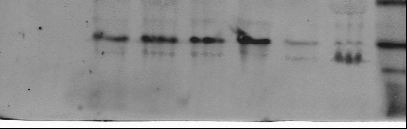





## Fig3C


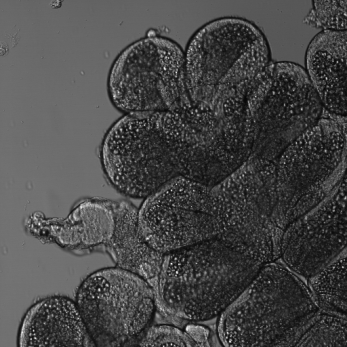

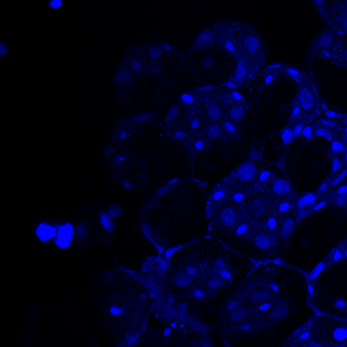

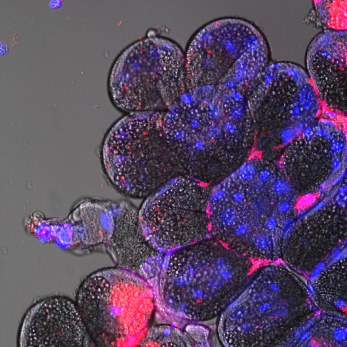

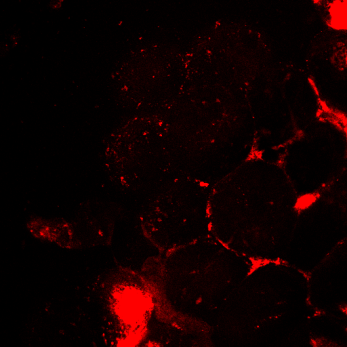


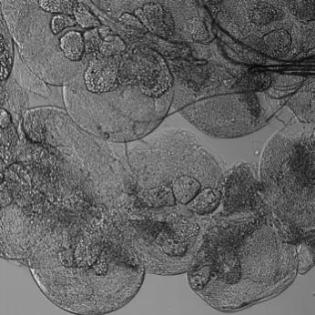

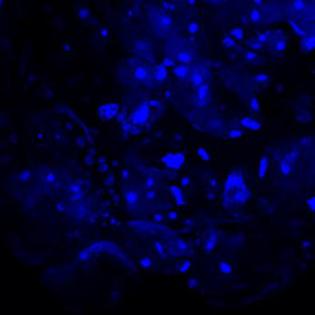

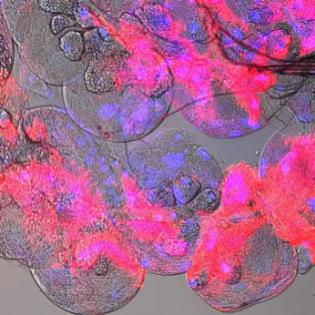

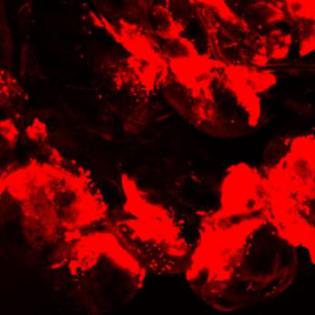


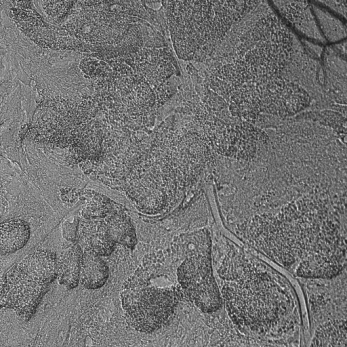

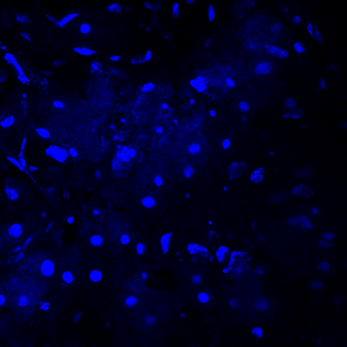

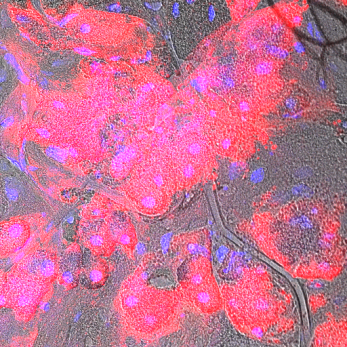

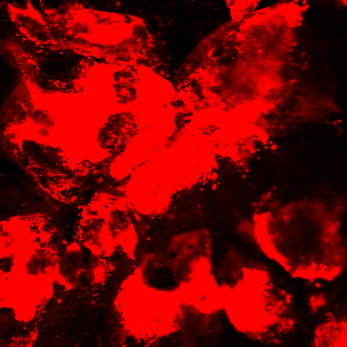


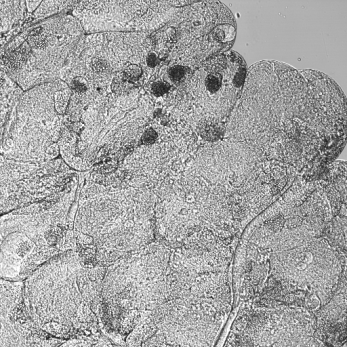

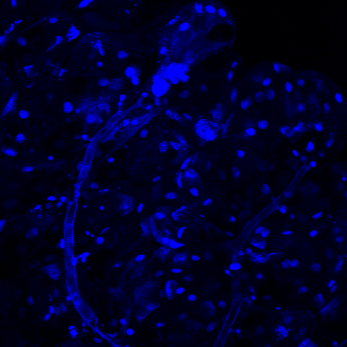

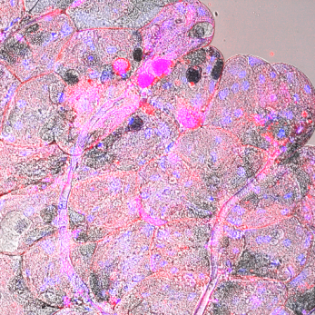

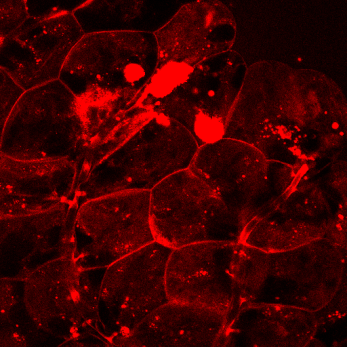


## Fig4A


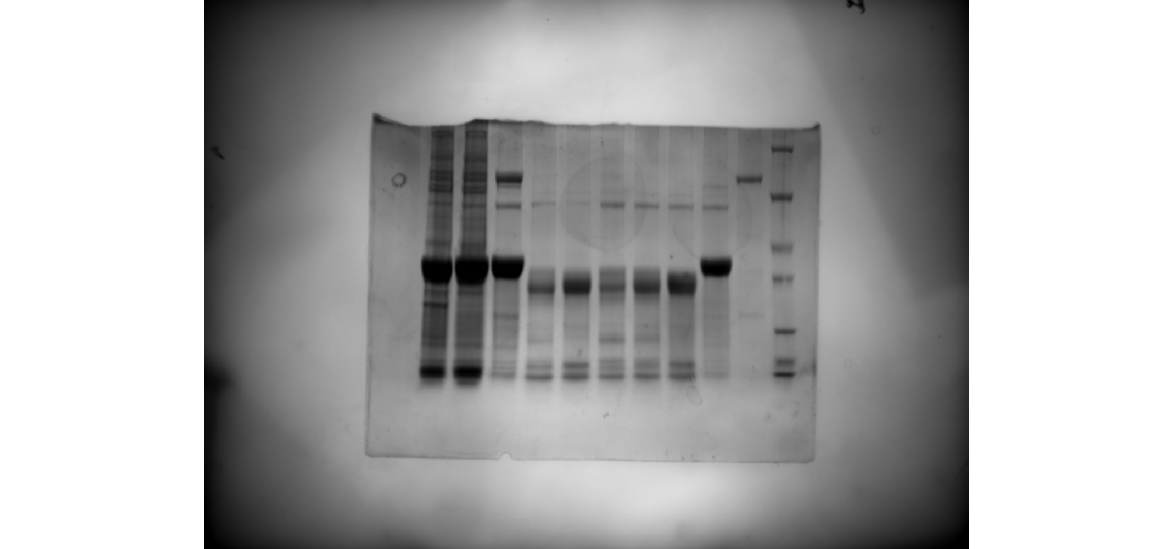


## Fig4B


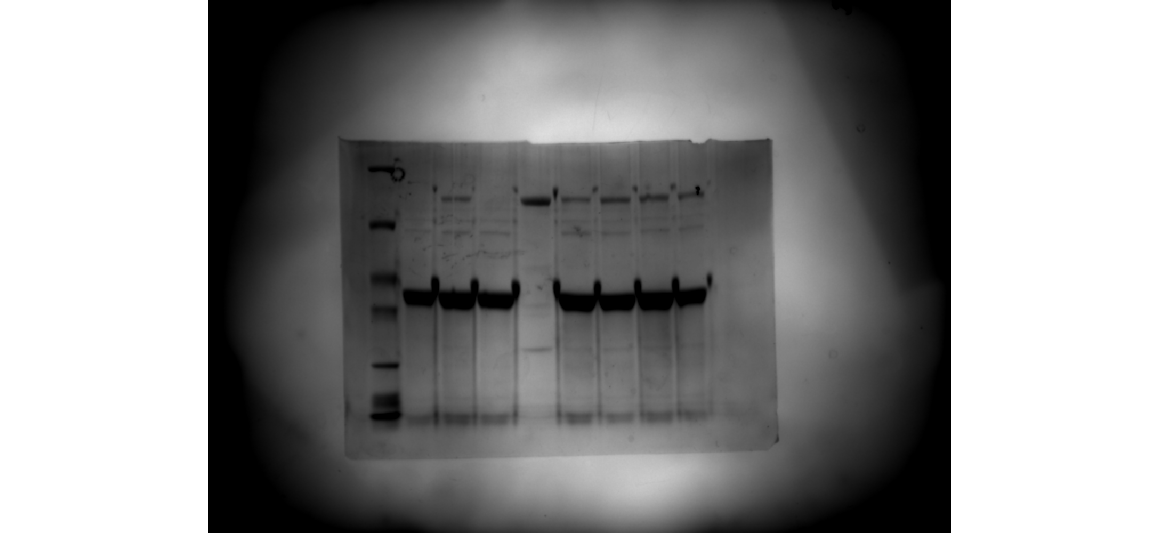


## Fig5A


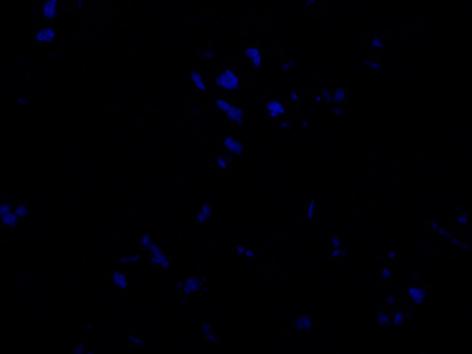

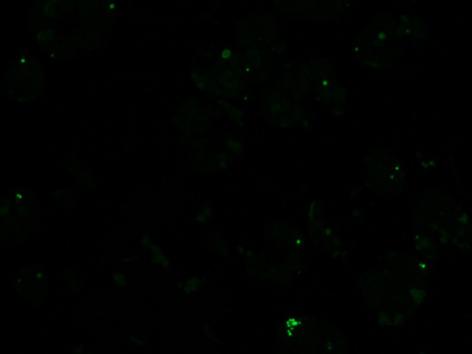

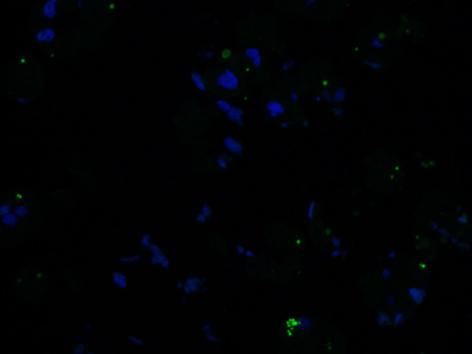

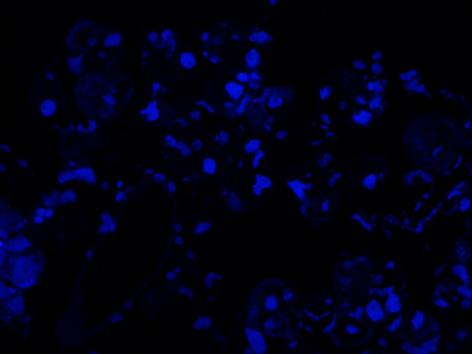

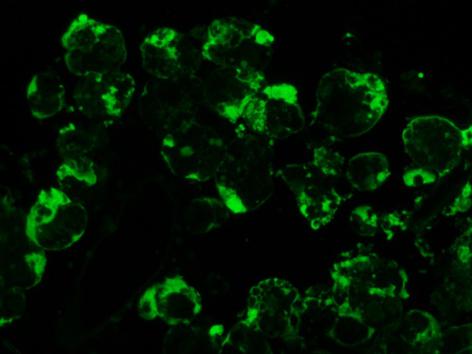

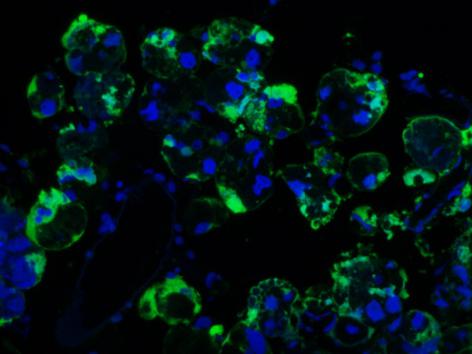

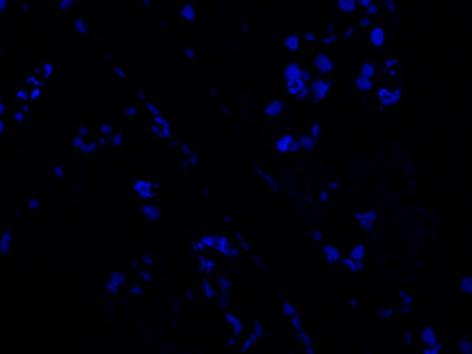

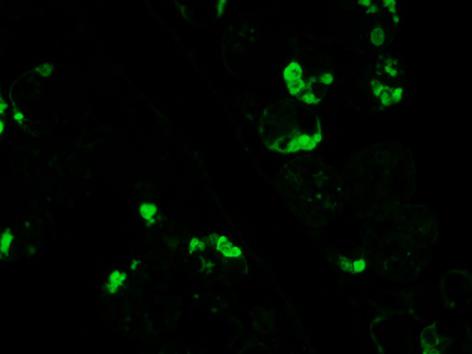

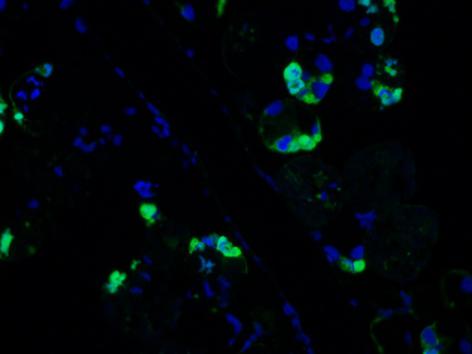


## Fig5B


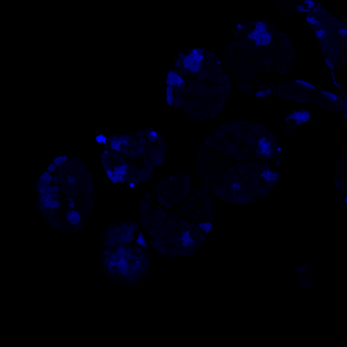

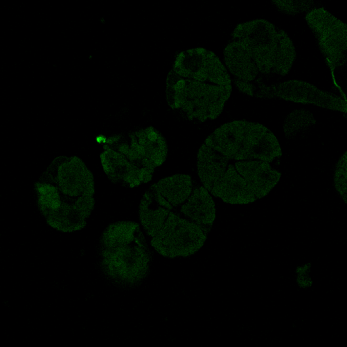

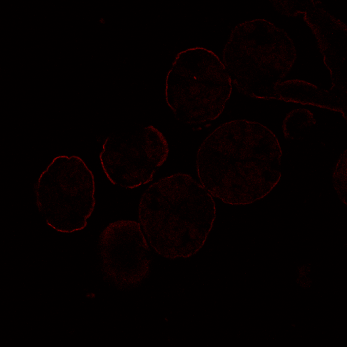

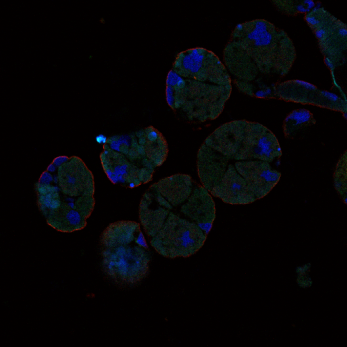


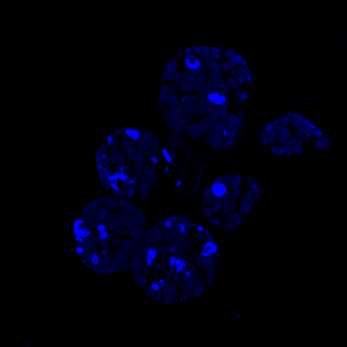

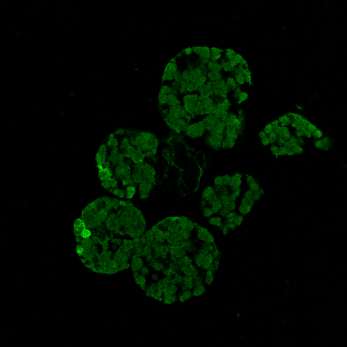

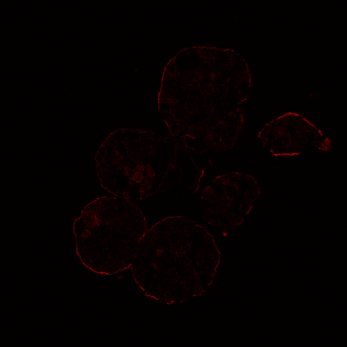

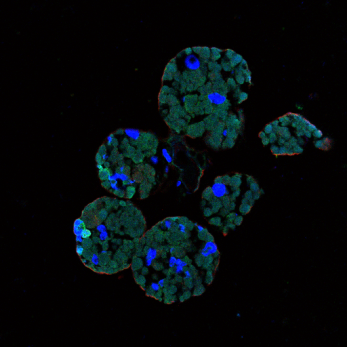


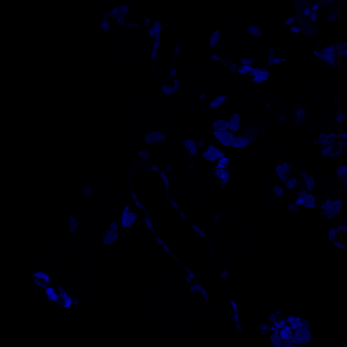

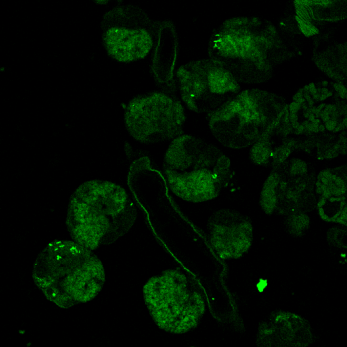

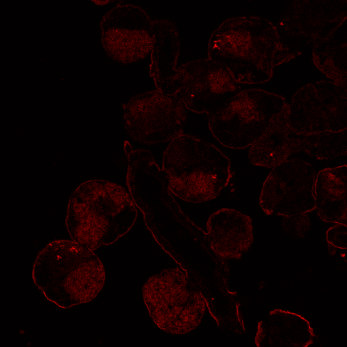

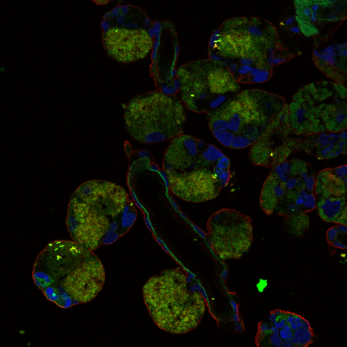


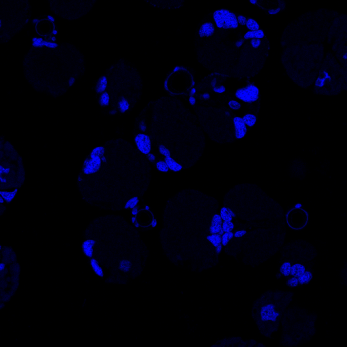

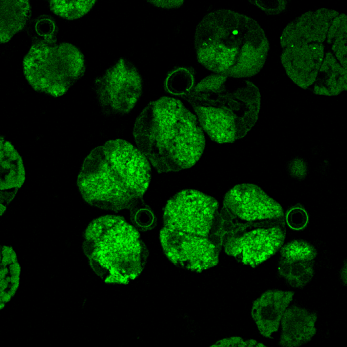

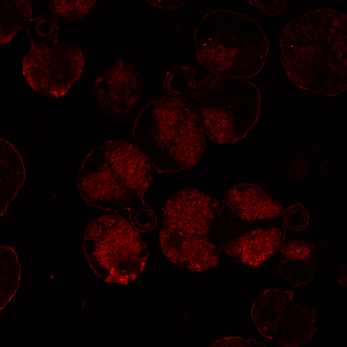

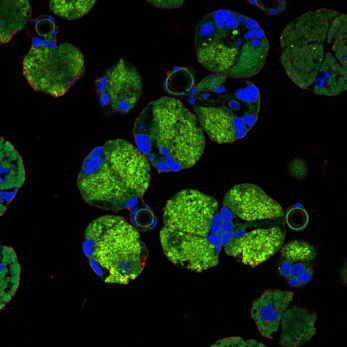


## Fig7A











## Fig7C


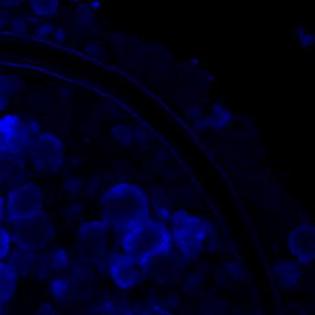

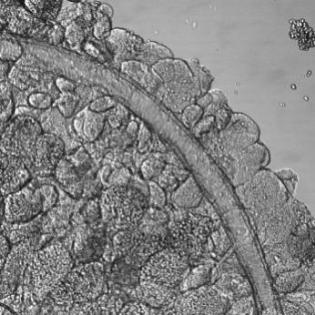

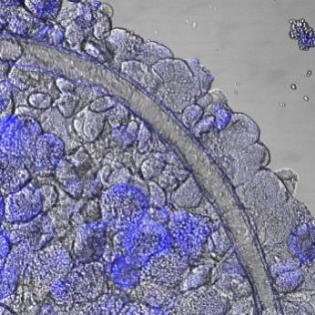

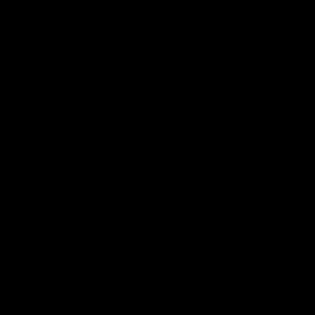


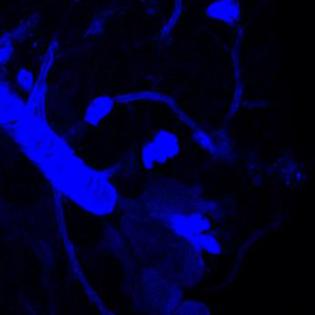

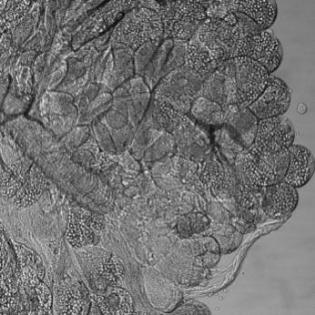

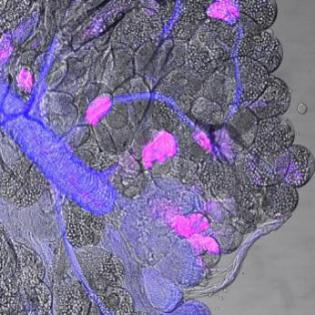

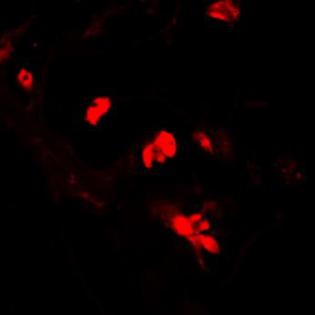


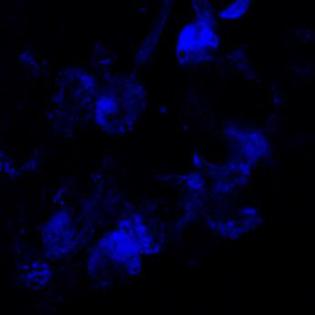

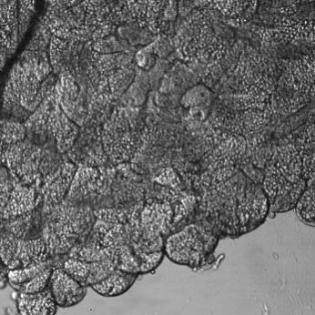

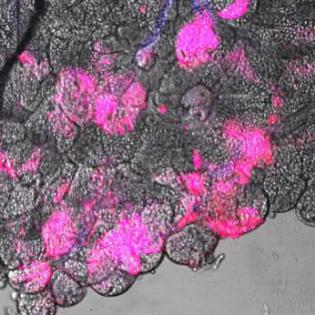

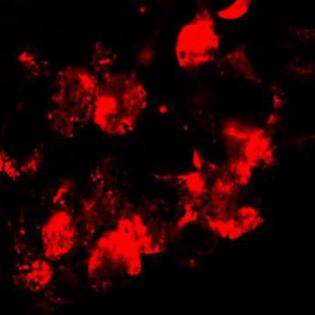


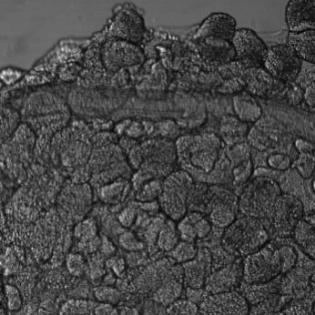

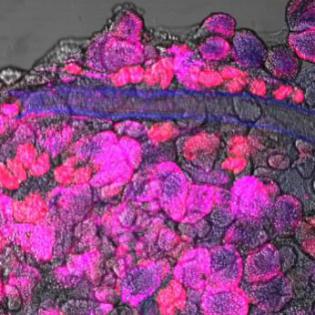

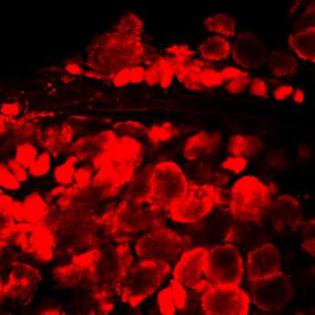

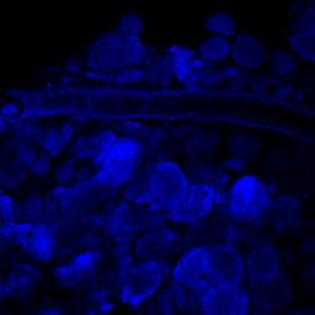


## Fig8A


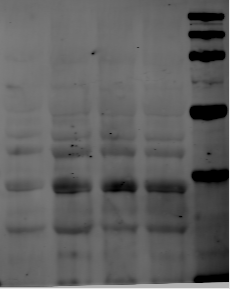

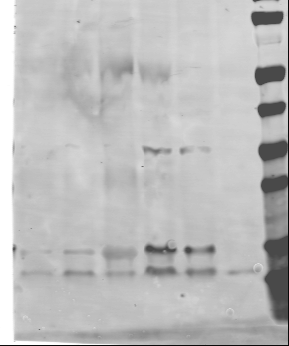

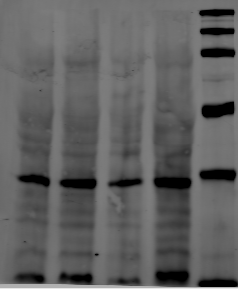

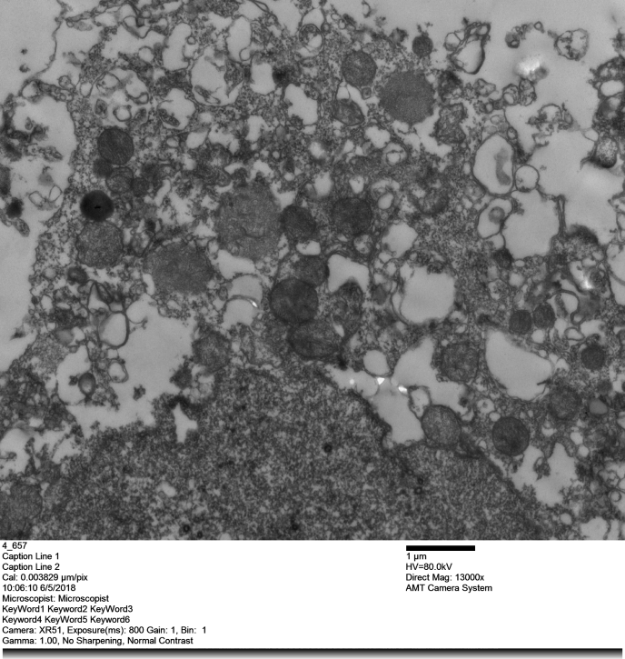

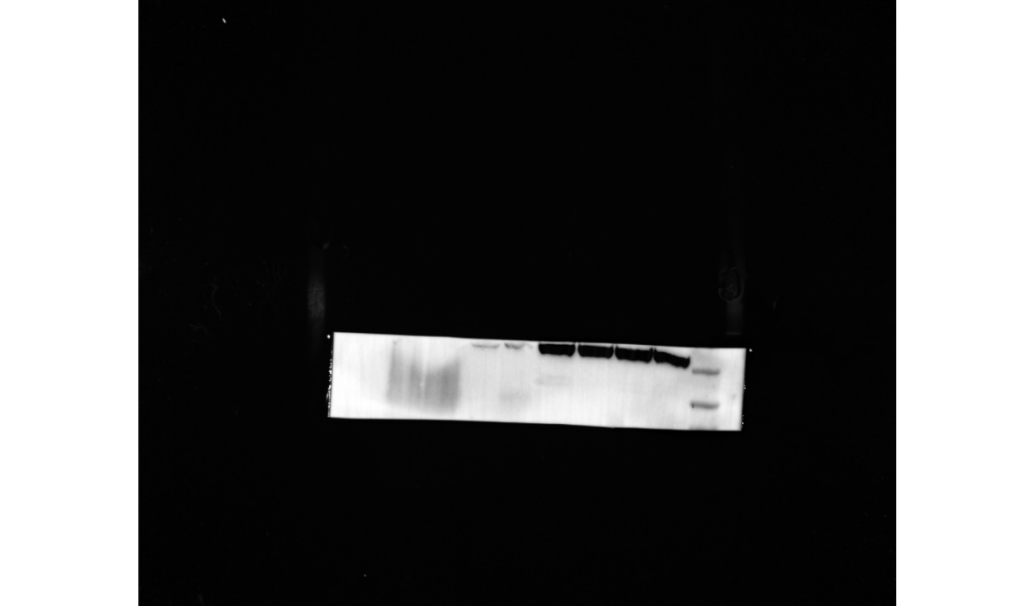


## Fig8B

## Fig8C

Supplement: S2 Data — (DOCX) [file pntd.0009074.s002.docx]
