# Supplementary material for: ATG5 is instrumental in the transition from autophagy to apoptosis during the degeneration of tick salivary glands
Source: PLoS Negl Trop Dis. 2021 Jan 29;15(1):e0009074. doi: 10.1371/journal.pntd.0009074 (PMC7875341; doi:10.1371/journal.pntd.0009074)
Supplement: S2 Table — S, forward primer; A, reverse primer; Sm, forward primer for deletion mutation; Am, reverse primer for deletion mutation; the sequence in bold underlined indicates the sequence of the T7 promoter. (DOCX) [file pntd.0009074.s006.docx]

**S2 Table** Primers for apoptosis and autophagy related gene cloning and vector construction

| **Primer name** | **Primer sequence** |
| --- | --- |
| RhATG5-S | CGCAGGTTCGATGTAAACATTTCTGTTC |
| RhATG5-A | AGACTAGCCGTAGAGTTTGAGAAATATCAC |
| RhATG5-pET-30a-S | GGCTGATATCGGATCCATGGCGGAAGATCGTGAAGTCTTAC |
| RhATG5-pET-30a-A | GTGCGGCCGCAAGCTTGGTTGTCTGGGTAACTGAGGTGTTCA |
| RhATG5^191-199Δ^-pET-30a-Sm | TTCAAGCACATACCTTTTCGCCTGAGGCTAGTCACGC |
| RhATG5^191-199Δ^-pET-30a-Am | GTGTCAATGGCGTGACTAGCCTCAGGCGAAAAGGTA |
| RhATG5 dsRNA-S1 | **GGATCCTAATACGACTCACTATAGG**CAAGGTCCATAAGCACTTCTCCAGG |
| RhATG5 dsRNA-A1 | CTCATCCATTGAAGCGGTGTGTCCA |
| RhATG5 dsRNA-S2 | CAAGGTCCATAAGCACTTCTCCAGG |
| RhATG5 dsRNA-A2 | **GGATCCTAATACGACTCACTATAGG**CTCATCCATTGAAGCGGTGTGTCCA |

**^a^**S, forward primer; A, reverse primer; Sm, forward primer for deletion mutation; Am, reverse primer for deletion mutation; the sequence in bold underlined indicates the sequence of the T7 promoter.
