# Supplementary material for: ATG5 is instrumental in the transition from autophagy to apoptosis during the degeneration of tick salivary glands
Source: PLoS Negl Trop Dis. 2021 Jan 29;15(1):e0009074. doi: 10.1371/journal.pntd.0009074 (PMC7875341; doi:10.1371/journal.pntd.0009074)
Supplement: Attachment — Submitted filename: Responses to the comments.docx [file pntd.0009074.s007.docx]

Responses to the comments

We are grateful to the editors for the constructive comments and suggestions that helped us to improve our paper. As indicated in the responses that follow, we have thoroughly taken each comment and suggestion into consideration in revising our files.

Comment#1-**Methods**
-Are the objectives of the study clearly articulated with a clear testable hypothesis stated?
-Is the study design appropriate to address the stated objectives?
-Is the population clearly described and appropriate for the hypothesis being tested?
-Is the sample size sufficient to ensure adequate power to address the hypothesis being tested?
-Were correct statistical analysis used to support conclusions?
-Are there concerns about ethical or regulatory requirements being met?

Re: Because few studies on autophagy and apoptosis in ticks are reported [1-7], we studied the research progress and methods in mammals and insects [8-15]. In this study, we selected the Ixodes ticks species *Rhipicephalus haemaphysaloides*, which is widely distributed in China, as the main experimental subjects. According to the biological characteristics of *R. haemaphysaloides*, we explored the appropriate experimental methods, sample collection and time point selection [7, 16-19]. In the study, our experimental methods, statistical analysis and animal experiment ethics are in a line with various requirements.

Comment#2-**Results**
-Does the analysis presented match the analysis plan?
-Are the results clearly and completely presented?
-Are the figures (Tables, Images) of sufficient quality for clarity?

Re: We re-checked all the statistical analysis data in the article and added new appropriate statistical analysis to make it better. For the figures, according to the requests, we uploaded all of them to the PACE tools and met the PLOS requirements.

Comment#3-**Conclusions**
-Are the conclusions supported by the data presented?
-Are the limitations of analysis clearly described?
-Do the authors discuss how these data can be helpful to advance our understanding of the topic under study?
-Is public health relevance addressed?
Re: all the data supported our conclusions. The lack of progress in the related field of ticks and the limitations of experimental methods sometimes make it impossible for us to fully verify the results (Lack of mature protein over-expression and gene knockout system). We revised our discussion to meet the results and topic better.

Comment#4 What's the different roles in ATG5 and ATG8 after 20E treated? The conclusion is not very clear.

Re: we added related contents in the discussion (line481-487).

ATG8 family protein plays a key role in autophagy. Proteins in this family, including microtubule-associated protein light chain 3 (LC3) and GATE-16 (GABARAPL2), are incorporated into the autophagosome membrane under autophagic stimulation [20]. Like other family members, GABARAP is cleaved at its carboxy terminus, which in turn leads to conjugation via phospholipid phosphatidylethanolamine or phosphatidylserine (PE) [21, 22]. ATG8-PE was identified as a maker molecular of autophagy [23-25].

The other type of important ubiquitinated protein is mainly composed of ATG5-ATG12. In addition to being an important autophagy molecule, ATG5 can also regulate the transformation of autophagy to apoptosis through NtATG5 [8, 9, 12].

Comment#5. The author must supply new clean WB of Fig3A, it's very hard to conclude from this WB plot, because they use polyclonal antibody, how they can distinguish the band of RhATG5/NtRhATG5 and RhATG8/PE? May need shRNA or KO data to support?

Re: we have tried many times, and always obtained similar results, it is difficult to get a better version due to the pcAb resistance. The following results still not in a good version. Considering that we decided made no changes to Fig3A in the article, and we uploaded the original figures.

ATG8-PE

Tublin

ATG8

fed-3d

fed-5d

fed-7d

engorged-1d

engorged-3d

engorged-5d

ATG5

pro-caspase3

Cleaved-caspase3

NtATG5

The results of western blot showed the specific of pcAb of RhATG5.

Before preparing the pcAb of RhATG8, we conducted an analysis of the amino acid sequence and found that the molecule has a homology of up to 92% and 84% with homo-GABARAP (UniProt ID: [O95166](http://www.uniprot.org/uniprot/O95166)) and homo-GABARAP L (UniProt ID:  [Q9H0R8](http://www.uniprot.org/uniprot/Q9H0R8)). We synthesized peptides of residues near the amino terminus as antigens for the preparation of pcAb (indicated by Cell Signaling Technology), so we believe that the band recognized by the polyclonal antibody is credible.

Comment#6. Figures need to be rearranged to 6-7 figs based on the result part.

Re: we re-edited the results for Figure 6-7 accordingly (line 393 and line 403-407).

Comment#7. Introduction part needs to be improved.

Re: we re-edited the introduction accordingly (line 82-89).

Comment#8. The authors must show more data of their RNAi experiments, it seems failed to RNAi from Fig8A.
Re: for the RNAi *in vitro*, qRT-PCR assays were detected for further verification (Fig S2).

Material and Methods:
Comment#9. Please describe with more details tick feeding, including number of ticks and rabbits used, time used for SG collection, etc.

Re: we added related the related contents in methods (line 146-147; line 816).

Comment#10. Did authors perform an efficiency curve for primers used for qPCR?

Re: we didn’t perform the efficiency curve for the primers, but we can provide the melt curve plot for the primers.

Comment#11. Line 204: Does “fed” mean fully engorged ticks? Please clarify.

Re: we are sorry for our mistakes, only unfed tick salivary glands were cultured *in vitro*.

Comment#12. Salivary glands were cultivated and treated with 20E at µM concentration. What is the concentration of 20E in tick hemolymph? Would be informative to discuss this.

Re: we added related the related contents in discussion from line 471 to line 487.

Comment#13. For RNAi and dsRNA synthesis, please describe the strategy used to avoid off-targets.
Re: we first maintain the specificity of the dsRNA synthesis primers, and then use different dsRNAs for pre-experiment. After qRT-PCR detection, we select the dsRNA with the highest interference efficiency for subsequent experiments.

Results:
Comment#14. Figure 1: DAPI seems to be staining salivary gland cytoplasm at 3 day-fed.

Re: As the TEM results showed below, there are a lot of mitochondria in tick salivary glands (especially in the fed tick salivary glands). Therefore, some mitochondria may be stained by DAPI.

**Fed-3d	Fed-5d	Engorged	Engorged-2d**

Comment#15. Authors should include a BLAST analysis against the new 6 high-quality genomes described recently (https://www.cell.com/cell/pdf/S0092-8674(20)30931-4.pdf). Would be informative to have such comparison with different tick species regarding these salivary gland genes described here.
Re: We have read this latest article on the tick genome. So far, we can’t retrieve the corresponding mRNA sequence of the annotated related tick species from the Internet. And according to the comments, we added some more ATG5 homologous genes of ticks for the evolutionary tree analysis (Fig.S1).

Comment#16. Please include statistical analysis for results described in Figure 2 (as done for Figure 6).
Re: according to your requests, we added related statistical analysis for Figure 2 and Figure 3A.

Comment#17. I would suggest authors to include as supplemental material full images of gels and WB.
Re: according to your requests, we have uploaded all the original figures as supplemental material.

Comment#18. Figure 4: Band intensity looks weak for a 10-20 µg load of calpain. Authors stated there is only one cleavage site for calpain and that RhATG5 was cleaved into one active fragment, however there are several bands (at least 3) resulted from this cleavage. 
Re: in the cleavage assays of RhATG5, our results showed that even the proRhATG5 appeared as towed belts, so we believe that it is reasonable for other two belts (NtATG5 and C-RhATG5). As the results showed that the reduction of RhATG5 resulted in a single belt.

Minor comments
Comment#19. Line 32: Please spell out ATG. There is no need to use capital letter for caspase (check all over the text).
Comment#20. Line 34: autophagy-related.
Comment#21. Line 177: isoelectric point
Comment#22. Line 225: (MKFQYKEEHPFEK).
Comment#23. Line 230: Missing a parenthesis after China.
Comment#24. Line 240: anti-alpha
Comment#25. Line 294: a total of
Comment#26. Figure S1: Please italicize species description
Comment#27. Line 333 and 334: autophagy and apoptosis.

Re: according to the comments, we revised all the mistakes above.
--------------------
Reference

1.	Umemiya-Shirafuji R, Matsuo T, & Fujisaki K Autophagy in ticks. Methods Enzymol. 2008; 451: 621-638. <http://www.ncbi.nlm.nih.gov/pubmed/19185742> PMID: 19185742

2.	Umemiya R*, et al.* Autophagy-related genes from a tick, Haemaphysalis longicornis. Autophagy. 2008; 4: 79-81. <http://www.ncbi.nlm.nih.gov/pubmed/17938584> PMID: 17938584

3.	Umemiya R*, et al.* Cloning and characterization of an autophagy-related gene, ATG12, from the three-host tick Haemaphysalis longicornis. Insect Biochem Mol Biol. 2007; 37: 975-984. <http://www.ncbi.nlm.nih.gov/pubmed/17681237> PMID: 17681237

4.	Ullah SA & Kaufman WR Salivary gland degeneration and ovary development in the Rocky Mountain wood tick, Dermacentor andersoni Stiles (Acari: Ixodidae). II. Determination of the 'critical weight'. Ticks Tick Borne Dis. 2014; 5: 516-522. <http://www.ncbi.nlm.nih.gov/pubmed/24865927> PMID: 24865927

5.	Tanaka M*, et al.* Molecular cloning of two caspase-like genes from the hard tick Haemaphysalis longicornis. J Vet Med Sci. 2007; 69: 85-90. <http://www.ncbi.nlm.nih.gov/pubmed/17283408> PMID: 17283408

6.	Alberdi P*, et al.* Tissue-Specific Signatures in the Transcriptional Response to Anaplasma phagocytophilum Infection of Ixodes scapularis and Ixodes ricinus Tick Cell Lines. Front Cell Infect Microbiol. 2016; 6: 20. <http://www.ncbi.nlm.nih.gov/pubmed/26904518> PMID: 26904518

7.	Yu X*, et al.* Caspase-1 participates in apoptosis of salivary glands in Rhipicephalus haemaphysaloides. Parasit Vectors. 2017; 10: 225. <http://www.ncbi.nlm.nih.gov/pubmed/28482931> PMID: 28482931

8.	Yousefi S*, et al.* Calpain-mediated cleavage of Atg5 switches autophagy to apoptosis. Nat Cell Biol. 2006; 8: 1124-1132. <https://www.ncbi.nlm.nih.gov/pubmed/16998475> PMID: 16998475

9.	Xie K*, et al.* BmATG5 and BmATG6 mediate apoptosis following autophagy induced by 20-hydroxyecdysone or starvation. Autophagy. 2016; 12: 381-396. <http://www.ncbi.nlm.nih.gov/pubmed/26727186> PMID: 26727186

10.	Kim M*, et al.* Mutation in ATG5 reduces autophagy and leads to ataxia with developmental delay. Elife. 2016; 5: <http://www.ncbi.nlm.nih.gov/pubmed/26812546> PMID: 26812546

11.	Li YB*, et al.* The steroid hormone 20-hydroxyecdysone upregulates calcium release-activated calcium channel modulator 1 expression to induce apoptosis in the midgut of Helicoverpa armigera. Cell Calcium. 2017; 68: 24-33. <http://www.ncbi.nlm.nih.gov/pubmed/29129205> PMID: 29129205

12.	Li YB, Li XR, Yang T, Wang JX, & Zhao XF The steroid hormone 20-hydroxyecdysone promotes switching from autophagy to apoptosis by increasing intracellular calcium levels. Insect Biochem Mol Biol. 2016; 79: 73-86. <http://www.ncbi.nlm.nih.gov/pubmed/27777042> PMID: 27777042

13.	Lee CY & Baehrecke EH Steroid regulation of autophagic programmed cell death during development. Development. 2001; 128: 1443-1455. <https://www.ncbi.nlm.nih.gov/pubmed/11262243> PMID: 11262243

14.	Kametaka S, Matsuura A, Wada Y, & Ohsumi Y Structural and functional analyses of APG5, a gene involved in autophagy in yeast. Gene. 1996; 178: 139-143. <https://www.ncbi.nlm.nih.gov/pubmed/8921905> PMID: 8921905

15.	Yousefi S & Simon HU Apoptosis regulation by autophagy gene 5. Crit Rev Oncol Hematol. 2007; 63: 241-244. <http://www.ncbi.nlm.nih.gov/pubmed/17644402> PMID: 17644402

16.	Wang Y*, et al.* Initiator and executioner caspases in salivary gland apoptosis of Rhipicephalus haemaphysaloides. Parasit Vectors. 2020; 13: 288. <https://www.ncbi.nlm.nih.gov/pubmed/32503655> PMID: 32503655

17.	Wang F*, et al.* Ovary Proteome Analysis Reveals RH36 Regulates Reproduction via Vitellin Uptake Mediated by HSP70 Protein in Hard Ticks. Front Cell Infect Microbiol. 2020; 10: 93. <https://www.ncbi.nlm.nih.gov/pubmed/32211346> PMID: 32211346

18.	Wang F*, et al.* Molecular characterization, tissue-specific expression, and RNA knockdown of the putative heat shock cognate 70 protein from Rhipicephalus haemaphysaloides. Parasitol Res. 2019; 118: 1363-1370. <https://www.ncbi.nlm.nih.gov/pubmed/30891634> PMID: 30891634

19.	Yu X*, et al.* Differential sialotranscriptomes of unfed and fed Rhipicephalus haemaphysaloides, with particular regard to differentially expressed genes of cysteine proteases. Parasit Vectors. 2015; 8: 597. <https://www.ncbi.nlm.nih.gov/pubmed/26577685> PMID: 26577685

20.	Shpilka T, Weidberg H, Pietrokovski S, & Elazar Z Atg8: an autophagy-related ubiquitin-like protein family. Genome Biol. 2011; 12: 226. <https://www.ncbi.nlm.nih.gov/pubmed/21867568> PMID: 21867568

21.	Sou YS, Tanida I, Komatsu M, Ueno T, & Kominami E Phosphatidylserine in addition to phosphatidylethanolamine is an in vitro target of the mammalian Atg8 modifiers, LC3, GABARAP, and GATE-16. J Biol Chem. 2006; 281: 3017-3024. <https://www.ncbi.nlm.nih.gov/pubmed/16303767> PMID: 16303767

22.	Kabeya Y*, et al.* LC3, GABARAP and GATE16 localize to autophagosomal membrane depending on form-II formation. J Cell Sci. 2004; 117: 2805-2812. <https://www.ncbi.nlm.nih.gov/pubmed/15169837> PMID: 15169837

23.	Chakrama FZ*, et al.* GABARAPL1 (GEC1) associates with autophagic vesicles. Autophagy. 2010; 6: 495-505. <https://www.ncbi.nlm.nih.gov/pubmed/20404487> PMID: 20404487

24.	Vernier-Magnin S*, et al.* A novel early estrogen-regulated gene gec1 encodes a protein related to GABARAP. Biochem Biophys Res Commun. 2001; 284: 118-125. <https://www.ncbi.nlm.nih.gov/pubmed/11374880> PMID: 11374880

25.	Pellerin I*, et al.* Identification and characterization of an early estrogen-regulated RNA in cultured guinea-pig endometrial cells. Mol Cell Endocrinol. 1993; 90: R17-21. <https://www.ncbi.nlm.nih.gov/pubmed/8495796> PMID: 8495796
